# Murine Model Imitating Chronic Wound Infections for Evaluation of Antimicrobial Photodynamic Therapy Efficacy

**DOI:** 10.3389/fmicb.2016.01258

**Published:** 2016-08-09

**Authors:** Grzegorz Fila, Kamola Kasimova, Yaxal Arenas, Joanna Nakonieczna, Mariusz Grinholc, Krzysztof P. Bielawski, Lothar Lilge

**Affiliations:** ^1^Laboratory of Molecular Diagnostics, Department of Biotechnology, Intercollegiate Faculty of Biotechnology, University of Gdansk and Medical University of GdanskGdansk, Poland; ^2^Princess Margaret Cancer Centre, University Health NetworkToronto, ON, Canada; ^3^Theralase Inc., TorontoON, Canada; ^4^Department of Medical Biophysics, University of TorontoToronto, ON, Canada

**Keywords:** photodynamic therapy, topical infection, chronic wounds, bioluminescence, *Pseudomonas aeruginosa*, methicillin resistant *Staphylococcus aureus*

## Abstract

It is generally acknowledged that the age of antibiotics could come to an end, due to their widespread, and inappropriate use. Particularly for chronic wounds alternatives are being thought. Antimicrobial Photodynamic Therapy (APDT) is a potential candidate, and while approved for some indications, such as periodontitis, chronic sinusitis and other niche indications, its use in chronic wounds is not established. To further facilitate the development of APDT in chronic wounds we present an easy to use animal model exhibiting the key hallmarks of chronic wounds, based on full-thickness skin wounds paired with an optically transparent cover. The moisture-retaining wound exhibited rapid expansion of pathogen colonies up to 8 days while not jeopardizing the host survival. Use of two bioluminescent pathogens; methicillin resistant *Staphylococcus aureus* (MRSA) and *Pseudomonas aeruginosa* permits real time monitoring of the pathogens. The murine model was employed to evaluate the performance of four different photosensitizers as mediators in Photodynamic Therapy. While all four photosensitizers, Rose Bengal, porphyrin TMPyP, New Methylene Blue, and TLD1411 demonstrated good to excellent antimicrobial efficacy in planktonic solutions at 1 to 50 μM concentrations, whereas in *in vivo* the growth delay was limited with 24–48 h delay in pathogen expansion for MRSA, and we noticed longer growth suppression of *P. aeruginosa* with TLD1411 mediated Photodynamic Therapy. The murine model will enable developing new strategies for enhancement of APDT for chronic wound infections.

## Introduction

Chronic wounds, generally comprising diabetic foot ulcers, pressure ulcers, venous leg ulcers, burn wounds, and wounds “older than 3 months of age” (Kirketerp-Møller et al., 2011) are a worldwide healthcare issue. They are causing a cycle of pain, anxiety and reduced quality of life for the individual patient, and present a considerable cost to the health care providers and patient (Dowsett, 2015). It is estimated that chronic wound therapies account for 2–4% of the total health system expenses, including material cost, nurse time, and hospitalization, that estimate is excluding the indirect costs such as loss of productivity and out-of-pocket expenses for patients (Sen et al., 2009). Over 90% of chronic wounds contain bacteria and fungi acquired from the skin, oral mucosa, enteric tract, or the environment (Kucera et al., 2014), which prevent tissue remodeling and healing. These bacteria can form a multispecies biofilm, which is often held responsible for the further development of an infection (Kucera et al., 2014). Infections commonly benefit from a priori compromised immune functions due to other diseases and/or their treatments, resulting in the lack of particular immune system components such as B cells, T cells, antibodies, neutrophils, damage to immune organs etc. Secondary immunodeficiency is often exploited by various microorganisms leading to grievous infections. Immunodeficiency is a well-established risk factor for cancer patients receiving chemotherapy (Pardoll, 2012; Khan et al., 2015).

Any break in the barrier function of the skin and other epithelial layers, predisposes an individual to infections (Schreier and Chatterjee, 2015). One of the major issues in controlling the healing initiation versus infection is sustaining the moisture balance in the tissue. Swollen and suppurative wounds provide perfect growth conditions for anaerobic and aerobic pathogens colonization and expansion thereof (Kirketerp-Møller et al., 2011), whereas a dehydrated wound surface can delay the healing process. Hence, while preserving a moist wound environment is advisable (Lilge et al., 2000) concurrent eradication and suppression of pathogenic microorganisms are highly desirable. A delay or failure in the treatment of moist wounds can lead to bacterial colonization progression and can cause the development of systemic infection.

Currently, there are some therapies to control infected wounds. The most classical – antibiotic therapy – starts to be inefficient as multidrug-resistant strains keep spreading and continue to expand unchecked. The increase in multidrug resistant strains to conventional antibacterial therapies has prompted the development of alternatives antimicrobial therapies, particularly for hard-to-heal wounds (Troxler et al., 2006). Negative pressure wound therapy promotes wound healing by applying a vacuum through a special dressing. It increases the granulation tissue formation and the local blood flow and enhances the bacterial clearance (Ene et al., 2015). Wound dressing saturated with silver, iodine or other antimicrobial agents can help the body achieve the ideal moist, warm healing conditions and simultaneously protect the wound from environmental exposure during this process (Murphy and Evans, 2012). Numerous plants are used in folk medicine against various diseases, albeit the exact mechanism of their action remains largely unknown, although antimicrobial properties of this phytotherapy have been investigated (Halcon and Milkus, 2004; Joubert et al., 2008; Kumar et al., 2008) and many of them are considered biofilm disruption agents. Combination of ethnopharmacology and antibiotic therapy could provide an effective bactericidal tool for the treatment of various bacterial and yeast infections (Taraszkiewicz et al., 2013). Low-Level Laser Therapy (LLLT) seems to exert possibly exploitable antimicrobial effects, especially against *Pseudomonas aeruginosa* caused infections, whereas it appears to be less or ineffective against other bacterial strains (Nussbaum et al., 2003, 2009).

To address the local nature of chronically infected wounds Antimicrobial Photodynamic Therapy (APDT) provides appe- aling properties. In APDT, a chemical compound (photo- sensitizer) absorbs light photons with sufficient quantum energy resulting in its electronic excited state, from which energy can be transferred to biomolecules or to molecular oxygen. The latter leads to the generation of Reactive Oxygen Species (ROS) or singlet oxygen formation, which can cause cell damage and microorganism’s death as recently reported by Taraszkiewicz et al. (2013) in *in vitro* studies of APDT. Localization of the therapy is achieved by two different mechanisms; first the confinement of the activation light to the clinical target area and second the difference in temporal photosensitizer association and uptake by bacteria versus host cells.

While efficacy of APDT has been demonstrated in various planktonic *in vitro* studies, transition to *in vivo* studies showed variable results, with some pre-clinical models showing good efficacy, whereas the majority of the studies demonstrated a poor translation of the APDT efficacy *in vivo*.

Typically, to quantify a therapy’s efficacy, based on the eradicated bacterial number, one group of hosts need to be sacrificed immediately after treatment and a second group need to be kept up to 50 h to determine the microbiota survival fraction. Blood samples can also be taken to analyze possible bacteremia development. Hamblin et al. (2003) and Wong et al. (2005) created full thickness excisional wounds, by using surgical scissors and forceps. Small wounds (8–9 mm × 12.5 mm) were infected with bioluminescent bacterial strains *P. aeruginosa* (10^6^) or *Vibrio vulnificus* (10^6^), allowing observation of wound healing and infection development within individual animals. As wounds remain uncovered, mice had to be kept separate to prevent opportunistic contaminations between animals. Zolfaghari et al. (2009) used two wound models: excisional and superficial. In the first case, shaved and depilated skin was pinched by sterile forceps and a 6 mm circular area (28 mm^2^) was cut down till the subcutaneous areolar tissue using scissors. In the second model a 25 mm^2^ square shaped wound was created by scarification using a 27G needle. Both skin preparations were inoculated with 10^8^ CFUml^-1^ MRSA cells on the wounds. To assess the effectiveness of the therapy mice were sacrificed upon treatment and the number of surviving bacteria was quantified following incubation on agar plates.

The ideal pre-clinical model of hard-to-heal wounds should be based on an immunocompromised host, thus permitting systematic infection. However, that model requires weeks to be established and carries significant risk of sepsis and survival failure. Also the use of immunocompromised animals can dramatically increase the cost of the research. Minimal animal number, wound drying, dressing changes causing wound oxygenation variations, unnecessary animal pain, or large surface area damage are some of the important factors to be considered during the *in vivo* experimental design stage.

Here we demonstrate a Tegaderm^TM^ based topical infection model for antimicrobial PDT as local antimicrobial therapy, and demonstrate its utility by testing Rose Bengal (RB), porphyrin TMPyP New Methylene Blue (NMB), or TLD1411 mediated APDT.

By the use of strong skin adhering, vapor and light transmitting dressings (Lilge et al., 2000), a moist wound environment is maintained providing perfect condition for wound healing as well as bacterial growth and the development of infection. Dressings can be kept for several days, isolating wounds from environmental- and cross-contaminations, permitting co-housing of animals thus reducing their stress level. Of additional advantage is the use of bioluminescent bacterial strains permitting non-invasive longitudinal monitoring of the therapeutic efficacy in each animal, reducing the number of animals required to achieve statistical significance. Moreover, from an animal care point, the possibility to decrease the wound size as well as provide opportunistic infection prevention will promote better tissue reconstruction.

To demonstrate the utility of the murine model, we validated the efficacy of 4 different photosensitizers RB, porphyrin TMPyP, NMB, and TLD1411 as APDT mediators in planktonic solution, demonstrated the need for a covered wound for maintaining an ongoing infection and finally demonstrated the ability to quantify APDT outcome in this murine model, *in vivo* as time delay until pathogen regrowth is noted.

## Materials and Methods

### Animals

All procedures were approved by the Animal Care Committee (ACC) of University Health Network, Toronto, ON, Canada (protocol number AUP 3303.2). FVB/N mice of both sexes, originally obtained from Xenogen Corporation – Alameda, CA but tested negative for the luciferase gene were used. Animals were housed in 12-h day/night cycles, at 20°C room temperature, granulated food, and water were provided ad libitum.

In total 51 mice were required for this study and randomly assigned to one of 6 groups: (i) non-infected mice (*n* = 3) to establish the duration of normal wound healing. Groups (ii) to (iv) represent infected wounds without APDT comprising: (ii) Tegaderm dressing, (iii) no dressing, and (iv) removal of dressing at the end of day 2 from established infection to evaluate the need for the transparent dressing to maintain the infection control throughout the observation period. For these groups *n* = 3 animals were used for each of the two bacterial strains employed here. The APDT treatment groups v) comprised *n* ≥ 3 × 2 bacterial strains for each of the four photosensitizers were tested. Group (vi) evaluated APDT efficacy versus potential LLLT effects employing a light only group for both bacterial strains.

### Infected Wound Induction Procedure

The day before wound establishment, the animals were anesthetized by isoflurane using 4% for induction and 1.5 to 2.5% for maintenance delivered via nose cone. Their dorsal surface hair was shaved and remaining hair removed with depilatory lotion. The next day, after repeating anesthesia a pinch of skin in the center of the shaved area (0.5 cm to 1 cm above the ilium bones) was held by tweezers and top layer of the skin is cut off to cause 5 to 6 mm diameter wound.

Immediately after cutting the skin the wound area was inoculated with10 μl of PBS containing 10^7^ CFU bacteria using a pipette tip. For mice in groups ii to vi Tegaderm^TM^ was applied immediately as per group assignment and kept for the assigned time.

### Dressing

1.5 cm by 1.5 cm squares of Tegaderm^TM^ (Transparent Film Roll 3M^TM^) were cut and applied to the wound area. According to commercial specification, the dressing is breathable permitting oxygen in and moisture vapor out thus allowing the skin to function normally. Tegaderm^TM^ is transparent and no light attenuation needs to be considered (Lilge et al., 2000). Tegaderm^TM^ studies have demonstrated positive effect on wound healing process in patients. Patients reported less pain during dressing changes, as well as more comfort with daily usage (Ravenscroft et al., 2006).

### Bacterial Strains

Two bioluminescence pathogens: Methicillin Resistant *Staphy- lococcus aureus* (MRSA) strain (XEN) (Caliper, City, State) and *Pseudomonas aeruginosa* (PAK) were used. PAK is a wild type, commonly studied *P. aeruginosa* strain containing and expressing a full complement of virulence factors. The strain has *luxAB* inserted into its chromosome thereby exhibiting bioluminescent properties (Ramphal et al., 2008).

Bacteria were incubated for 18 h at 37°C in fresh LB liquid broth. After overnight incubation, tubes were vortexed and the volume adjusted to obtain 10^7^ CFUml^-1^. Next, bacteria were centrifuged and re-suspended in 20 μl sterile PBS. To control the concentration of bacteria a serial dilutions of the utilized suspension was prepared and inoculated on fresh LB agar plate followed by incubation for 18 h at 37°C. Colonies were counted on the next day to determine the original bacteria concentration.

### Light Source

A custom built LED light source emitting λ = 525 ± 15 nm (manufactured by Theralase Inc. Toronto, ON, Canada) was employed and the output was focused on the wound area. The light source of non-coherent light, delivering an irradiance of 50 mWcm^-2^ to the wound surface as verified by a NIST traceable optical power meter (Nova Power Meter, Ophir Photonics). The light source was used previously for *in vivo* phototherapy studies and did not cause thermal effects (Arenas et al., 2013; Fong et al., 2015). The light was administrated continuously for 37 min, resulting in a radiant exposure of 100 Jcm^-2^.

### Photosensitizers

Four Photosensitizers were evaluated for their APDT efficacy *in vitro* and *in vivo*: RB, NMB, TMPyP, and TLC1411. All photosensitizers were excited at 525 nm to minimize APDT efficacy differences due to variations in the wavelength dependent penetration depth into the moist infected wounds. Comparing the efficacy between the photosensitizers is thus only a function between their respective molar extinction coefficients at 525 nm.

Rose Bengal (4,5,6,7-tetrachloro-2′,4′,5′,7′-tetraiodofluores- cein) (Sigma–Aldrich) is a sodium salt used in medicine as an eye drops to stain damaged conjunctival and corneal cells (Ervin et al., 2010). Maximum absorption wavelength λ_max_ = 548 nm and the molar extinction coefficient at 525 nm is 3653 molL^-1^cm^-1^. The singlet oxygen quantum yield was reported as 0.74 (Lee and Rodgers, 1987).

New Methylene Blue is a thiazine class compound used as a dye or antimicrobial agent (Ragas et al., 2013) with an absorption maximum at λ_max_ = 590 nm, a molar extinction coefficient at 525 nm of 471 molL^-1^cm^-1^ and a singlet oxygen quantum yield of 0.66 ± 0.04 in air equilibrated acetonitrile (Ronzani et al., 2013).

TMPyP [5,10,15,20-Tetrakis(1-methyl-4-pyridinio) porp- hyrin tetra(p-toluenesulfonate)] (Sigma–Aldrich) is a positively charged, two-photon dye, that can bind to DNA in cell cultures and affect the cell viability. Its one photon peak absorption wavelength λ_max_ = 421 nm. Its molar extinction coefficient at 525 nm is 1306 molL^-1^cm^-1^, and it has a singlet oxygen quantum yield of 0.74 (Reddi et al., 2002).

TLD1411 is a Ru(II) coordination complex of the form [Ru(2,2′ -bipyridine)2(2-(2′,2″:5″,2″′-terthiophene)-imidazo[4,5-f][1,10] phenanthroline)]2+, originally synthesized by Dr. McFarland at Arcadia University and provided by Sigma Aldrich Fine Chemicals (Milwaukee, WI, USA). Its use as an APDT agent has been previously described (Arenas et al., 2013). Its absorption maximum is 430 nm in water and its molar extinction coefficient at 525 nm is 831 molL^-1^cm^-1^. The singlet oxygen quantum yield was reported as >0.9 in complete media (Shi et al., 2015).

**FIGURE 1:**
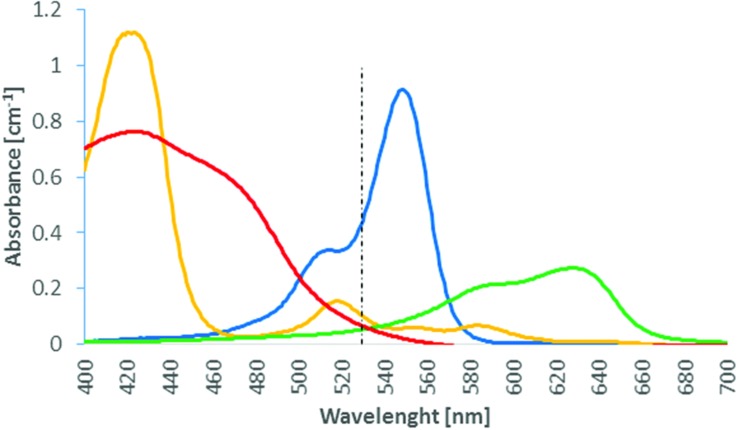
**Absorption coefficient of 10 μM solutions of Rose Bengal (blue), New Methylene blue (NMB) (green), TMPyP (orange), and TLD1411 (red) as function of wavelength.** The 525 nm Antimicrobial Photodynamic Therapy (APDT) activation wavelength is indicated.

**Figure 1** shows the wavelength resolved molar extinction coefficient of these four photosensitizers.

All photosensitizers were freshly prepared prior to the procedure as 1 mM stock solution in water, and diluted to the proper test concentration before being added to the bacterial culture.

### *In vitro* Experiments

All procedures were performed using sterile 96-well plates. Bacteria were diluted in fresh Luria–Bertani (LB) broth to a final planktonic concentration of 3⋅10^7^ CFUml^-1^. The photosensitizer solution was added at predetermined concentrations ranging from 1 μM to 150 μM equivalent to a final concentration of 2⋅10^8^ to 3⋅10^10^ PS molecules per bacterium. 100 μl of the PS solution was pipetted into wells followed immediately by photoactivation using the light source described above. Non-irradiated sample served as negative controls. Upon completion of light administration, samples were serial diluted and inoculated on fresh LB agar plates. A positive result required more than 3 magnitudes of log_10_ per CFUml^-1^ reduction in these planktonic conditions. PDT irradiation used a 96-diode laser array delivering 100 Jcm^-2^ total radiant exposure.

### *In vivo* Experiments

All procedures of wound establishment and bacterial inoculation were executed as described above. 30 min post bacterial inoculation 30 μl of the 500 μM photosensitizer solutions was injected under the dressing by Hamilton syringe and allowed to spread over the wound for an estimated final concentration of ~2⋅10^9^ photosensitizer molecules per bacterium. Photoactivation was initiated after 30 min of wait period.

### Bioluminescence Imaging

Images were collected using the IVIS Spectrum imaging system (Caliper Life Sciences, Hopkinton, MA, USA). For imaging, mice were anesthetized using isoflurane, as described above. Mice were imaged at specific time points: immediately after bacteria inoculation, prior to and after PS injection, prior to and after light administration, then every 10 min post APDT therapy up to 2–3 h, followed then daily up to 5th day or the endpoint determined in the protocols. APDT efficacy for bacterial inactivation was measured by loss of bioluminescent signal, defined as average radiance [hυs^-1^cm^-2^sr^-1^] and subjective evaluation of the wound’s appearance. The bioluminescence (BLI) radiance was determined by selecting equal size of the region-of-interest (ROI) directly over the wound and between the two shoulders blade to determine the cameras dark counts.

## Results

### Infection Development in Open versus Covered Wounds

**FIGURE 2:**
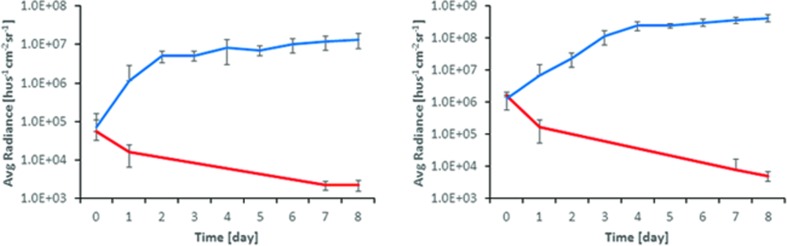
**Comparison of BLI signal from open (red) and Tegaderm^TM^ dressed (blue) wounds for (Left) *XEN* and (Right) *PAK***.

In Tegaderm^TM^ covered wounds an increase in bioluminescent signal was observed after inoculation with either PAK or XEN strain, starting from day 0 up to 3rd (PAK) or 4th (XEN) day of experiment. Then, a plateau phase was reached lasting up to 8th day after wound inoculation with bacteria (**Figure 2**). On the contrary, in wounds left open from the beginning as well as those where the Tegaderm^TM^ was removed after 48 h, a reversal of the initial increase in BLI radiance was observed. The wound’s appearance was dry after 2 days, and a pink appearance was retained.

**FIGURE 3:**
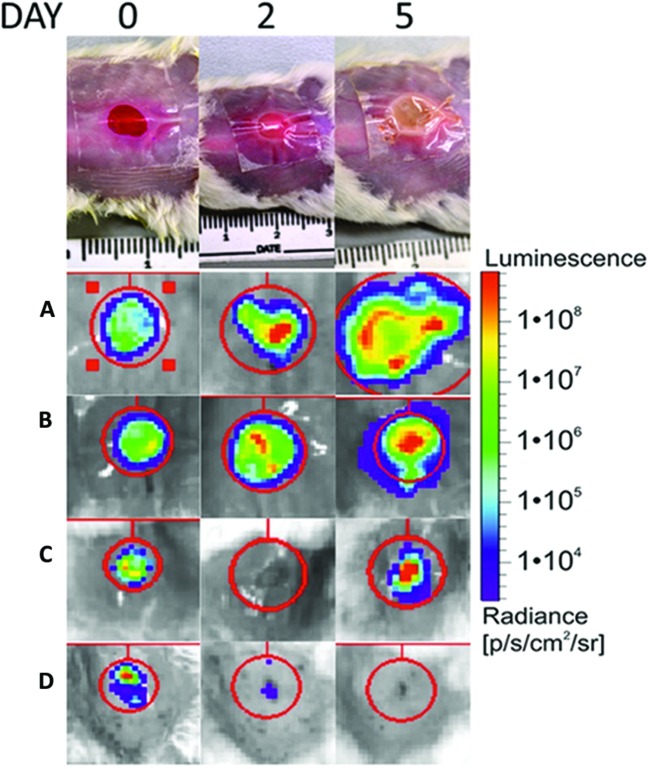
**Top shows the visual appearance of the wound over the 5-day observation period for a covered; non-treated wounds (A), covered, treated with Rose Bengal, *Pseudomonas aeruginosa* infected wound (B); covered; treated with TLD1411; *Staphylococcus aureus* infected wounds (C); uncovered, non-treated; infected wounds (D)**. The spread of the infection is clearly visible in (**A** and **B**), while **(C)** showed delay of infection. Left upper corners: Shown are examples of BLI signal as function of time over the first 5 days following wound generation and infection.

The appearance of the Tegaderm^TM^ dressed wound changed from the clear raw appearance to yellowish dense mucus on day 2 and 3, whereas they appeared yellow for the remainder of the observed duration. Often the wound expanded and the infection bulged the dressing outward as shown in **Figure 3**, for PAK inoculated wounds.

### APDT in Planktonic Solutions

**FIGURE 4:**
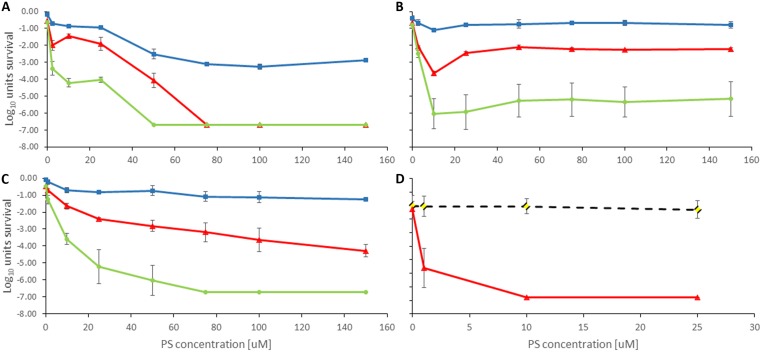
**Antimicrobial Photodynamic Therapy inactivation in planktonic solutions for PAK with (A) Rose Bengal, (B) NMB, (C) TMPyP, and (D) TLD1411.** The radiant exposure used is indicated as blue squares = 50 Jcm^-2^, red triangles = 100 Jcm^-2^, and green circles = 150 Jcm^-2^. The black dotted line with rhombus in **(D)** indicates dark toxicity.

**FIGURE 5:**
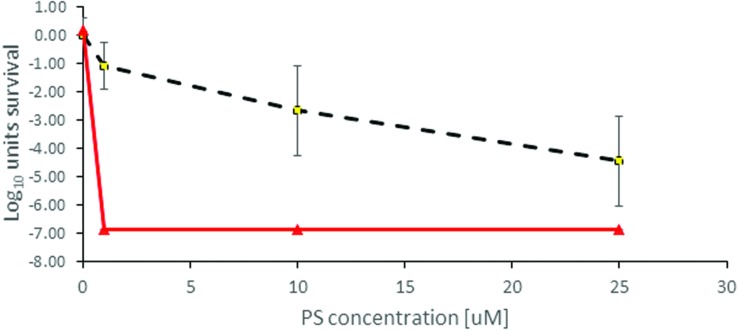
**Antimicrobial Photodynamic Therapy inactivation in planktonic solutions for *XEN* mediated by TLD1411.** The employed radiant exposure is indicated as black dotted line with rhombus = 0 Jcm^-2^, red triangles = 100 Jcm^-2^.

There was no significant dark toxicity of the photosensitizers toward the investigated bacteria within the concentration range studied (up to 500 μM), except for TLD1411 at >10 μM for XEN. Whereas, upon illumination all studied photosensitizers showed activity against PAK, and TLD1411 also against XEN bacteria; see **Figures 4** and **5**, for PAK and XEN results, respectively. The susceptibilities of bacteria to APDT mediated by one of the four sensitizers, was tested at increasing concentration of photosensitizers at three radiant-exposures ranging from 50 to 150 Jcm^-2^.

Rose Bengal showed positive effect toward PAK with 75 μM concentration and 50 Jcm^-2^ radiant exposure. Increasing light dose up to 150 Jcm^-2^ resulted in a decreasing RB concentration required to elicit a response. 50 μM of RB and 150 Jcm^-2^ decreased below the level of detection CFU ml^-1^ units up to 6 log_10_ (**Figure 4A**). APDT response reciprocity between radiant exposure and RB concentration breaks down for concentration above 50 μM presumably due to self-shielding within the 1 cm path length in the planktonic solutions used in these experiments, see below.

New Methylene Blue showed least antimicrobial activity against PAK. Only 10 μM concentration showed reduction of 3 log_10_ of survival cells when 100 Jcm^-2^ were applied, whereas 50 Jcm^-2^ failed to elicit a beneficial APDT response, both presumably due to self-shielding. At higher doses the photosensitizer showed no significant impact on efficacy. Unlike Rose Bengal, NMB had no dose-dependent efficacy, thus no significant difference in PAK inactivation from 10 to 150 μM when exposed the solution to 150 Jcm^-2^ (**Figure 4B**).

Porphyrin TMPyP reached a positive anti-bacterial effect against PAK when 150 or 100 Jcm^-2^ were administered. Increasing the light exposure led to a higher inactivation effect at lower PS doses and even 10 μM concentration exhibit significant CFU ml^-1^ reduction. The combination of 75 μM concentration and 150 Jcm^-2^ of green light resulted in 6 log_10_ eradication of PAK (**Figure 4C**). As for NMB 50 Jcm^-2^ did not initiate sufficient PAK inactivation again due to self-shielding within the 1 cm path length cuvette. Self-shielding also caused loss of reciprocity between light and PS doses.

TLD1411 was tested only for 100 Jcm^-2^ against PAK, so more than 6 log_10_ was achieved at the lowest concentration of 1 μM (**Figure 4D**) effectively reaching sterilization levels.

Only TLD 1411 was tested for its activity against XEN in these studies, and similar to PAK, 6 log_10_ inactivation was achieved with the lowest concentration of 1 μM (**Figure 3**). However, while no significant dark toxicity against PAK was observed, TLD1411 showed significant dark toxicity, ~3 log_10_, at 10 μM against XEN as noted already above.

**Table 1** shows the critical concentration required to achieve 3 log_10_ and 6 log_10_ reduction in bacterial cell count for the highest radiant exposure tested at 525 nm.

**Table 1:** Phototoxic sensitizer concentrations.

Photosensitizer	PAK		XEN	
	3 log_10_	6 log_10_	3 log_10_	6 log_10_
Rose Bengal	10 μM	50 μM	N/A	N/A
NMB	10 μM	>150 μM	N/A	N/A
TMPyP	10 μM	75 μM	N/A	N/A
TLD1411	1 μM	10 μM	<1 μM	<1 μM

### *In vivo* APDT in Infected Wounds

**FIGURE 6:**
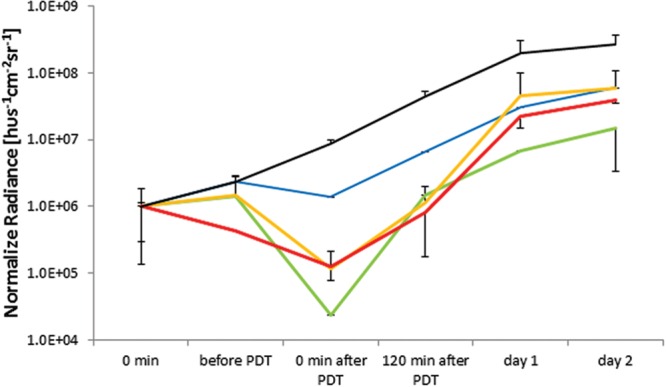
***In vivo* APDT bioluminescence for *PAK* in an untreated control (solid black), Rose Bengal (blue), NMB (green), TMPyP (orange), and TLD1411 (red).** The concentration of either PS was ~2⋅10^9^ molecules per bacterium.

Unlike for *in vitro* planktonic cultures, the *in vivo* response was much more moderate, with none of the photosensitizers achieving sterilization of the wound equivalent to a 6 log_10_ reduction. In fact, not even clearing at 3 log_10_ was attainable based on BLI radiance measurements, see **Figure 6**.

As there was a variation in the group average BLI radiance for PAK studies, the BLI signal was normalized at *t* = 0 just prior to PS administration, permitting comparison of the APDT effects.

Untreated wounds with expanding bacterial counts reached bioluminescence signal saturation at the first day. The two principal reasons for this saturation effect are increased infection thickness reabsorbing the bioluminescence photons and increased light scattering from the wound further confining the BLI radiance to the infection.

Rose Bengal mediated APDT resulted in the least BLI radiance reduction, ~ one order of magnitude, or ~ 3.2 pathogen division times, presenting limited benefit to wound management. NMB had the highest BLI radiance reduction following APDT (~ two orders of magnitude); however, the BLI radiance regain was faster than in untreated wound suggesting that the radiance loss was due to biochemical luminescence quenching or bleaching rather than bacterial inactivation. TMPyP had the 2nd best APDT induced BLI radiance reduction, whereas post APDT signal gains were comparable to untreated wounds suggesting true PAK inactivation or destruction by APDT. TLD1411 showed signs of dark toxicity prior to PDT with accelerated BLI radiance loss during PDT. BLI radiance recovery over the first 2 h was slower and accelerated over the next 22 h, reaching BLI radiance increase rate comparable to untreated control wounds.

Nevertheless, it should be noted that APDT mediated by either photosensitizer resulted in a lower BLI radiance intensity than non-treated control at 120 min, demonstrating the ability to delay PAK infection progression by APDT of induced wounds, see **Figure 6**.

**FIGURE 7:**
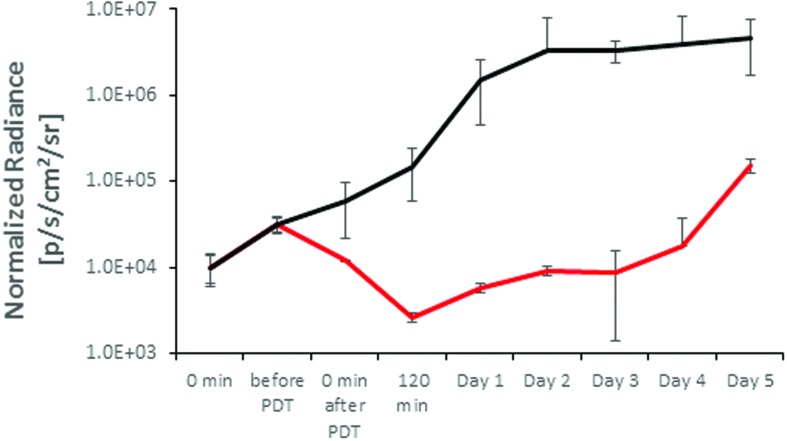
***In vivo* modulation of the *XEN* bioluminescence in an untreated control (solid black) and by TLD1411 mediated APDT (red)**.

The temporal evaluation of the XEN associated BLI in untreated controls and TLD1411 mediated APDT is shown in **Figure 7**, whereby untreated wounds reached BLI signal saturation at 2 days post infection. The TLD1411 concentration per XEN bacterium is estimated at ~2⋅10^7^ and photo-irradiation showed a continuous decrease in BLI radiance up to 90 min post PDT (120 min time point). The regain of BLI radiance was not significant until day 4, followed by a signal increase rate comparable to the untreated control.

Group vi, included for evaluation of APDT efficacy versus potential LLLT stimulatory effects due to light irradiation with both bacterial strains were identical to group ii (non-treated, infected wounds covered by Tegaderm). Bioluminescence radiance signal increased for 2 days, reaching 10^8^ and 10^6^ hυs^-1^cm^-2^sr^-1^ of average normalized radiance for PAK and XEN, respectively, and remained constant for the remainder of the observation period (data not shown). Hence, LLLT does not appear to be a confounder to PAK or XEN survival at 525 nm.

## Discussion

While various infected wound models have been published, there is still a need for an applicable model mimicking the clinical features of the disease, with short implementation time and efficient translation of the study results into clinical practice. Lee et al. (2011) created a large wound (1.5 by 2.5 cm) by tape-stripping and sandpaper based removing of stratum corneum and inoculating a bacterial suspension containing ~10^10^ CFUml^-1^ *S. aureus* cells. Biopsies were collected at specific time points to monitor wound healing and host cell growth. Mice were kept in individual cages to prevent cross contamination, affecting overall well-being of the animal by causing stress. Saito et al. (2012) exposed dorsal skin to 90°C to induce burn injury up to 20% of the total body surface area and then inoculated with 10^8^ CFU of *P. aeruginosa*. The model mimics large, festering wounds and can cause high pain and stress levels. Application of an occlusive dressing to avoid cross contamination is favorable in this model. Similar wounds were established by Hashimoto et al. (2012), where preheated steel was applied to the dorsal surface. Large burned areas (6–6.5 cm^2^) were immediately infected using 10^8^ *P. aeruginosa* cells.

The murine model of an infected wound presented here is robust and spontaneous bacterial clearing was not observed in the Tegaderm^TM^ covered wounds, thus it presents a favorable environment for testing APDT efficacy *in vivo*. The wound cover allows for oxygen and water vapor across the barrier and does not impede light penetration either for BLI, APDT, or LLLT (Lilge et al., 2000). It provides constant growth environment for both PAK and XEN, as demonstrated in **Figures 2** and **3**, but prevents opportunistic and environmental wound cross-contamination. Several mice can be housed in the same cage reducing stress levels during the experiment. Wounds, which were not covered or intentionally uncovered, dried out rapidly and infections were controlled without intervention either by the murine immune system or simple dying of the bacterium in an unfavorable environment possibly leading to an overestimation of the APDT efficacy.

Here the model was only utilized in the acute stage of infection, so sufficiently long to open the possibility of resilience against APDT, but insufficient to generate a deep infection, particularly because the pO_2_ in chronically infected wounds has been reported to be 30% to 90% lower than in healthy tissue (Schreml et al., 2010). The latter may require photosensitizers exploiting both type I and II APDT mechanisms (Fong et al., 2015; Shi et al., 2015).

However, the combination of BLI together with consistent growth environment allows testing various APDT protocols including the number of retreatment frequencies and extending the infection control.

As the APDT activation wavelength impacts the penetration depth in the *in vivo* model, the most common wavelength of 525 nm was selected. However, the varying molar extinction coefficient of these 4 photosensitizers led to self-shielding and possibly with that reason limited efficacy in planktonic solution for RB and NMB was observed. Selecting different concentrations to match the molar extinction coefficient would impact on the number of photosensitizers per bacterium, whereas selecting different wavelength to match the molar extinction coefficient at constant concentration would affect the light distribution in the tissue and hence by diffuse reflected light also the photon density at the tissue surface.

At time of APDT light treatment *in vivo* the target must be considered 2D with minimum full-thickness wounds and no observable presence of blood in an infected area. Hence, comparing the efficacy using this animal model system is valid purely on tissue optics consideration.

Nevertheless, the added attenuation of PS in *in vivo* condition needs to be considered, particularly when comparing PS efficacy in APDT. The worst-case impact on APDT radiance exposure on the target can be estimated based on the solution volume (30 μl) over the 6 mm diameter wound, which would result in an ~1 mm layer large compared to the diffusion distance of the ROS. As the PS acts also as a 525 nm light filter prior to the pathogens on the tissue surface, the resulting optical densities are RB = 1.83, NMB = 0.235, TMPyP = 0.653, and TLD1411 = 0.4155, respectively. In particular, for RB the high concentration employed in conjunction with the high molar extinction coefficient eventually caused a reduction of the radiant exposure at the microbes to 1.48 Jcm^-2^. Similarly, the worst-case radiant exposures for the other photosensitizers would be NMB = 58.2 Jcm^-2^, TMPyP = 22.2 Jcm^-2^, and TLD1411 = 38.4 Jcm^-2^, respectively. However, the solution was rapidly absorbed into the tissue and the filter effect by the photosensitizer had a lesser impact on the sensitizer specific APDT efficacy differences.

Conversely, trying to achieve a comparable OD in the tissue to eliminate effect of self-shielding as a confounding factor. There is a need for either adjustment of the photosensitizer’s concentration affecting the ratio of photosensitizing molecules per bacterium or selecting particular wavelength for each photosensitizer so that the molar extinction coefficients are the same, which in turn would impact the photon distribution in the tissue. Hence, our approach of using constant wavelength and radiant exposures in the sensitizer was compared.

To maintain reasonable penetration of the activated light into the tissue, the presence of oxy- or deoxy-hemoglobin needs to be considered as their absorption coefficients for 525 nm are > 10^2^ cm^-1^ and the penetration depth would be limited to < 100 μm (Jacques, 2013). Hence, the model can only be valid for wounds free of blood or scab. When treating burns a longer absorption wavelength is required for NMB or mixture of Ru(II) containing photosensitizer with carrying molecules, such as transferrin, which notably increases their molar extinction coefficient in the 600 to 800 nm range (Kaspler et al., 2016). Care must be taken regarding possible LLLT effects at these longer wavelength (Nussbaum et al., 2003).

A radiant exposure of 150 Jcm^-2^ can be achieved in 12.5 min according to the guidelines for safe irradiation of the skin, and the duration is also clinically acceptable. However, the experiments showed that for optimal efficacy of the therapy, a maximum irradiance of 50 mWcm^-2^ should not be exceeded allowing sufficient oxygen diffusion throughout the Tegaderm^TM^ wound cover. So care must be taken as possible photosensitizer reuptake into mammalian host cells, particular cells associated with the immune system needs to be considered when the irradiation times need to be extended significantly.

The efficacy of various photosensitizers against bacteria in planktonic solutions have been previously published for RB (Melo et al., 2011) and TMPyP (Cassidy et al., 2010) reporting concentration of 10 μmol/L (6 log of killing) and 250 μg/L (4 log of killing), respectively.

All photosensitizers have displayed good to excellent efficacy in bacterial inactivation for planktonic solutions particularly for PAK where 3 of 4 reached 6 log_10_ inactivation which is considered as sterilization state and the other photosensitizer (NMB) achieved at least 3 log_10_ of inactivation for 150 Jcm^-2^. The efficacy limits for RB and TMPyP are probably an artifact due to their high molar extinction coefficient and the high concentration used, which prevented effective illumination of the planktonic solution over the 1 cm sample depth. Radiant exposure with a much lower absorption coefficient of NMB compared to RB, its limited efficacy cannot be attributed to self-shielding. Additionally, as the photosensitizers have comparable singlet oxygen quantum yield one needs to conclude that NMB does not associate well with PAK. TLD1411 appears to present higher efficacy compared to other photosensitizers with a concentration less than 10 μM being sufficient for transient PAK sterilization at 100 Jcm^-2^. This may be explained by the ability of these ruthenium complexes to lead to DNA cleavage (Mari et al., 2014; Shi et al., 2015).

The high efficacy of these PSs *in vitro* is poorly translated into the *in vivo* applications, as previously noted (Amin et al., 2016). The reason for this efficacy loss can be explained by host factors as well as bacterial burden and the virulence of individual strains as previously noted (Hamblin et al., 2003) and reflects a more challenging environment also for the treatment of human superficial infections.

One caveat to these experiments is the small number (*n* = 4–5) employed for the different study groups. While this sample size is too small to allow statistical analysis of the results between photosensitizers, it is large enough to demonstrate an APDT effect and the report time line for BLI signal delay and time to signal saturation.

While only up to a 1.5 log_10_ reduction in BLI associated radiance was detected by PS mediated APDT *in vivo*, it may still be of clinical use. Based on the BLI radiance expression of the PAK and XEN growth curve, pre and post light exposure, it becomes evident that for certain protocols and conditions APDT treated wounds do not reach the BLI radiance intensity of the unexposed control for at least 24 h in case of PAK mediated infection and more than 4 days for XEN mediated ones. Despite the lack of complete response in wound sterilization this additional time increases the period to early onset of risk for local or systemic infection, thus providing the possibility to perform wound culture and antibiotic sensitivity testing, as about 90% of blood and other cultures are detected and identified within 48 h (Cockerill et al., 2004). Peptide nucleic acid fluorescence *in situ* hybridization (PNA FISH) methods are currently used for direct identification of selected *Staphylococcus, Enterococcus, Klebsiella*, and *Candida* species in less than 2 h (Shepard et al., 2008). While a 1 to 2 h delay for PCA determination of loss or the presence of genes encoding antibiotic resistance are available and appear reasonable, PCA will not describe functional protein and may be misleading, hence, generally antibiotic susceptibility testing commonly requires 1 to 3 days and hence a general infection control for 24 to 72 h is desirable.

## Conclusion

Our murine model of consistent local wound infection with *Staphylococcus aureus* (MRSA) and *P. aeruginosa* was similar to chronic wounds, without spontaneous clearing due to wound drying. The progress of the infection can be monitored real-time for bioluminescent bacteria, thus permitting the rapid evaluation of APDT agents and treatment protocols *in vivo* under physiologically relevant conditions for bacterial growth.

The *in vivo* evaluation of antimicrobial effect of photo- sensitizers responded less effectively compared to what were seen *in vitro*, an observation also reported by others; however, the potentially significant clinical reduction of the bacterial load remains apparently feasible, causing delay in full activation of inflammatory response and hence reducing the risk of developing sepsis.

The presented murine model is a suitable platform to evaluate the feasibility and safety of repeated cycles of PDT, based on clearance time of photosensitizer from host mammalian cells.

## Author Contributions

GF has performed *in vitro* experiments with the use of RB, TMPyP, and NMB against *P. aeruginosa*, as well as all *in vivo* procedures. He wrote the first draft of the manuscript. KK assisted and supported the *in vivo* experiments. YA performed the *in vitro* experiments. JN: participated in the conception and design of the work; advised on the experimental design and writing of the manuscript. MG participated in the conception and design of the work; drafted the manuscript and revised it critically for important intellectual content. KB participated in designing of the experiments and helped drafting manuscript. LL designed the experiments together with Drs. MG, JN and KB, supervised GF, KK, and YA on a daily basis and is the senior responsible author for the manuscript together with GF. He also acts as the corresponding author on this manuscript.

## Conflict of Interest Statement

The authors declare that the research was conducted in the absence of any commercial or financial relationships that could be construed as a potential conflict of interest.
